# Reefgenomics.Org - a repository for marine genomics data

**DOI:** 10.1093/database/baw152

**Published:** 2016-12-26

**Authors:** Yi Jin Liew, Manuel Aranda, Christian R. Voolstra

**Affiliations:** Division of Biological and Environmental Science and Engineering (BESE), Red Sea Research Center, King Abdullah University of Science and Technology (KAUST), Saudi Arabia

## Abstract

Over the last decade, technological advancements have substantially decreased the cost and time of obtaining large amounts of sequencing data. Paired with the exponentially increased computing power, individual labs are now able to sequence genomes or transcriptomes to investigate biological questions of interest. This has led to a significant increase in available sequence data. Although the bulk of data published in articles are stored in public sequence databases, very often, only raw sequencing data are available; miscellaneous data such as assembled transcriptomes, genome annotations etc. are not easily obtainable through the same means. Here, we introduce our website (http://reefgenomics.org) that aims to centralize genomic and transcriptomic data from marine organisms. Besides providing convenient means to download sequences, we provide (where applicable) a genome browser to explore available genomic features, and a BLAST interface to search through the hosted sequences. Through the interface, multiple datasets can be queried simultaneously, allowing for the retrieval of matching sequences from organisms of interest. The minimalistic, no-frills interface reduces visual clutter, making it convenient for end-users to search and explore processed sequence data.

**Database URL:** http://reefgenomics.org

## Introduction

Driven primarily by continuous reduction in sequencing costs and increasing availability of computing resources over the last decade, the genomes of several marine organisms e.g. *Amphimedon queenslandica* (1), *Acropora digitifera* (2), *Aiptasia pallida* (3), and *Hydra vulgaris* (4), and the transcriptomes of many others (5–8) have now been sequenced. However, a disconnect exists between what is submitted in the form of primary sequence data and what is available in the form of assembled and annotated data. While the majority of studies provide primary sequence data to public repositories, e.g. NCBI (National Center for Biotechnology Information), EBI (European Bioinformatics Institute), and DDBJ (DNA Data Bank of Japan), many studies elect not to upload assembled and annotated genomes or transcriptomes (all mRNAs expressed from the genes of an organism) to public sequence databases. Also, transcriptomic data tend to be more disparate, as illustrated by a recent 20-coral comparative metastudy (9) that used published primary data to infer the evolutionary success of reef-building corals. Although the sources of all data are cited, a web database to peruse, search via BLAST (Basic Local Alignment and Search Tool) (10), and download relevant sequence data were also provided for the convenience of the readers (http://comparative.reefgenomics.org).

To facilitate dissemination of similar data, we designed and host a website with simplicity in mind. We aim to provide an online platform for sharing assembled sequence data, and at the same time, facilitate access and retrieval of sequence files, simplify searches for related sequences among hosted data, and to enable the visual exploration of genomic features. We intend that the ease of access will facilitate further analyses and pave the way for other comparative studies using these and additional data, fostering collaborations and discovery within the marine biology community.

## Results

### Main landing page

**Figure 1.**
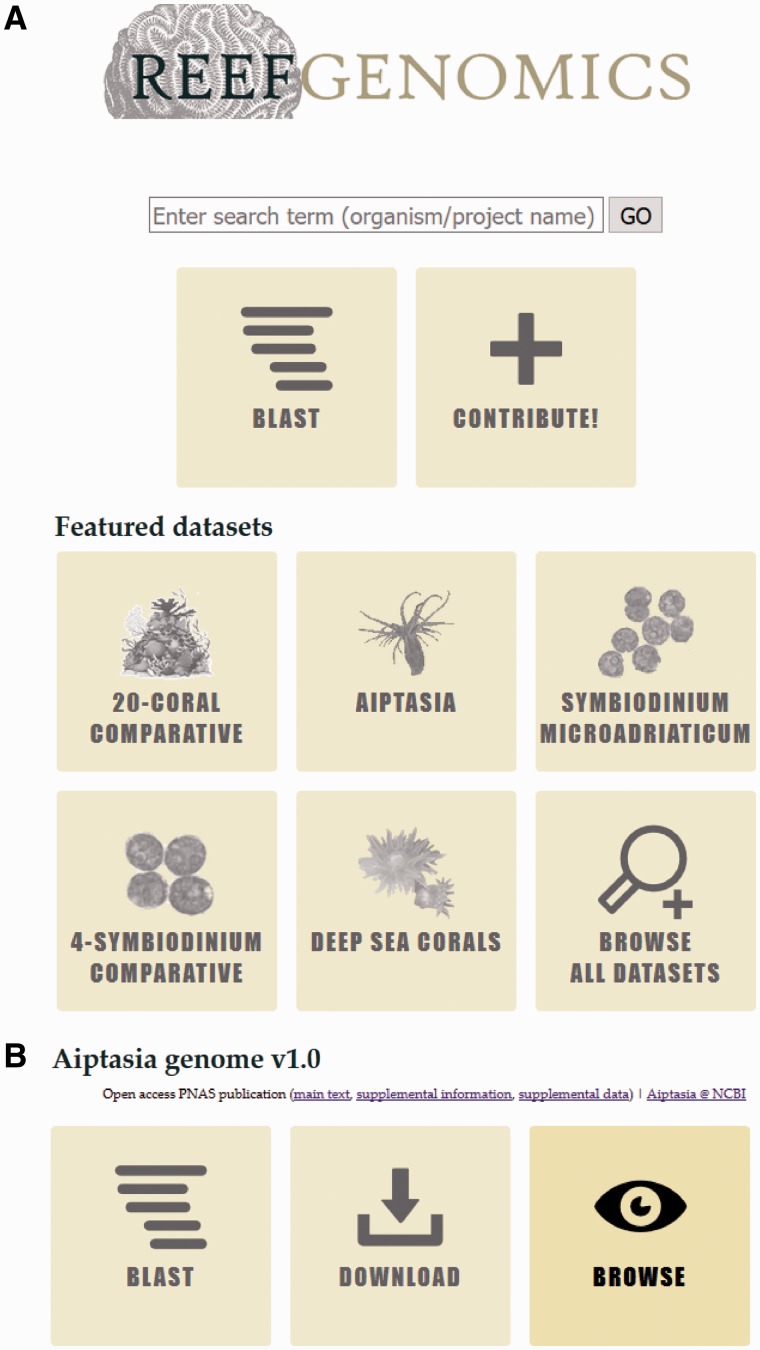
Layout of http://reefgenomics.org. **(A)** Landing page shows the search bar and tiles corresponding to featured projects and a tile to explore all datasets. **(B)** Project page shows the typical layout of a project-specific page. Hovering the cursor over tiles darkens them, as shown in the ‘Browse’ tile.

The main page (http://reefgenomics.org) has a text box - similar in design to a popular search engine - that allows new and experienced users alike to quickly jump to their organism(s) of interest. For new users that are unfamiliar with the datasets hosted on the website, we ease data discovery by featuring a collection of datasets, and we also provide a link to a complete list of all datasets on our website. Also on the main page is a prominent ‘Contribute’ button, which provides a point of contact for groups to contribute their data to our data portal (Figure 1A).

### Project-specific data sharing

From the main page, users are able to visit subdomains containing data produced by a project. At the time of writing, the hosted data range from individual genome projects, e.g. the genome of *Aiptasia* (3), to multi-institute collaborative efforts, e.g. a comparative study of 20 coral transcriptomes and genomes (9). Whenever possible, we opted to use short, memorable subdomain names instead of nested subdirectories. For instance, the *Aiptasia* genome project is located at http://aiptasia.reefgenomics.org; while the comparative study is at http://comparative.reefgenomics.org. Repeat users can quickly navigate to their organisms/datasets of interest by typing the memorable URLs in their browsers.

**Figure 2.**
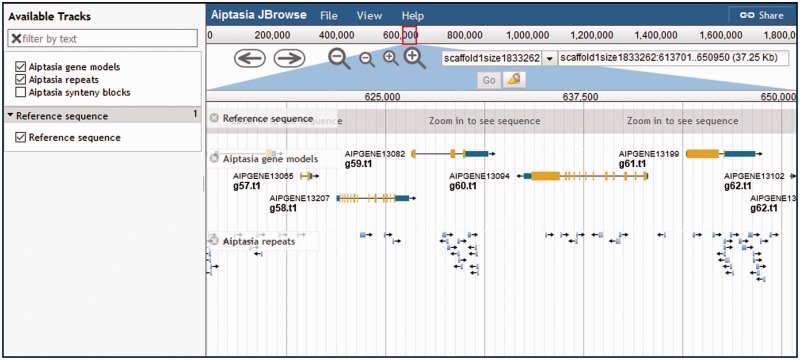
Visual exploration of genomic data accessible via the ‘Browse’ button. In this example, the repeats identified in the *Aiptasia* genome are overlaid on top of the gene models, allowing for a quick appraisal of genes with many repeat regions, and the relative position of these regions within the gene model.

Subdomains typically contain three buttons: ‘BLAST’, ‘Download’, and ‘Browse’ (Figure 1B). Some subdomains have fewer buttons depending on the hosted contents: for instance, transcriptome data cannot be viewed on a genome browser. The first button links users to a custom BLAST server based on SequenceServer (11), which produces aesthetically pleasing BLAST results that takes advantage of modern web standards. This BLAST server also has the advantage being able to search multiple databases simultaneously with a single query, which simplifies tasks, e.g. retrieving homolog for a particular gene of interest across many organisms. The ‘Download’ page is a typical HTML page that houses links to retrieve datasets, with a MD5 hash for users to verify that their datasets of interest were downloaded correctly. Last, the ‘Browse’ button allows for visual exploration of the hosted data via JBrowse (12). Data tracks can be added or removed via the sidebar on the left; relevant genes or scaffolds can be searched using the text box on the top-right, which supports autocompletion of features such as scaffold or gene names (Figure 2). Whenever applicable, we also provide links to the manuscript that produced/feature the data, as well as links to raw sequence data hosted on NCBI, effectively closing the loop from manuscript to raw data to processed data.

## Discussion

Currently, there are several other online portals with data from marine organisms: Metazome (https://metazome.jgi.doe.gov) (13), Compagen (http://compagen.org) (14), and OIST Genomic Projects (http://marinegenomics.oist.jp) (among others). Although there are similarities between reefgenomics.org and the other three websites, there are several features that distinguish our website from other data portals that are summarized in Table 1.

**Table 1.** Overview of features of other data portals with similarity to reefgenomics.org

Portal	reefgenomics.org	Metazome	Compagen	OIST genomic projects
**General features**				
**Scope**	Marine organisms	Metazoans	Early branching metazoans	Marine organisms
**Site organization**	By project	By organism	By organism	By organism
**Requires registration**	No	Yes	No	No
**Data availability**				
**Annotations**	Present	Present	Absent	Present
**Source**	Open for contributions	JGI-funded/JGI-linked projects	Open for contributions	OIST-funded projects
**Data exploration**				
**BLAST backend**	SequenceServer	Self-developed	wwwblast	Self-developed
**Search multiple databases?**	Yes	Yes	No	No
**Genome browser**	JBrowse	JBrowse	N/A	GBrowse

The reefgenomics.org data portal provides a clean and easy-to-use interface for interested researchers to access and explore data from marine organisms. Our intention is not to duplicate available sequence data, but to centralize data (on a project-specific basis) generated by multiple labs that are otherwise hard to access.

Arguably, the value and success of reefgenomics.org as a resource grows with the people that use it, the data deployed, and ensuring that the accessible content is up-to-date. Our hope is to promote growing community adaptation by providing an easily accessible and queriable database, facilitate hosting data for interested researchers, and by hosting data from larger projects within the framework of consortia, such as GIGA (the Global Invertebrate Genome Alliance) (15) and ReFuGe 2020 (Reef Future Genomics 2020) (16). Collaborative efforts that use reefgenomics.org as a central repository facilitate comparative analyses, while ensuring that this website will continue to be relevant and a central hub of ‘-omics’ data pertaining to marine organisms for the years to come.

## Methods

### Website hosting

This website is hosted on a Linode Cloud virtual server (https://www.linode.com) running Debian Stable and Apache 2. Linode also has the advantage of offering tiered plans for different usage requirements, making it convenient to scale the website up if it is under heavy use.

### Website design considerations

When designing the website, we coded it in a way to reduce dependencies and maximize compatibility with modern desktop browsers (Firefox, Chrome, and Edge). As such, whenever possible, the use of HTML5 and CSS3 was preferred over the use of large JavaScript libraries.

### Use of standard file formats

All genomic, transcriptomic, and proteomic data hosted on the website uses the standard FASTA format; genome feature annotations are in GFF3. Tabular data, e.g. annotations of individual genes, are provided as plain-text, tab-separated values to ease downstream parsing on the command-line and visual inspection via text editors or Microsoft Excel.
